# Eventive modal projection: the case of Spanish subjunctive relative clauses

**DOI:** 10.1007/s11050-023-09218-9

**Published:** 2024-02-19

**Authors:** Luis Alonso-Ovalle, Paula Menéndez-Benito, Aynat Rubinstein

**Affiliations:** 1 McGill University, Montreal, Canada; 2 University of Tübingen, Tübingen, Germany; 3 The Hebrew University of Jerusalem, Jerusalem, Israel

**Keywords:** Mood, Modality, Spanish, Subjunctive, Relative clauses, Event relativity

## Abstract

How do modal expressions determine which possibilities they range over? According to the Modal Anchor Hypothesis (Kratzer in *The language-cognition interface: Actes du 19*^*e*^ *congrès international des linguistes*, Libraire Droz, Genève, 179–199, 2013), modal expressions determine their domain of quantification from particulars (events, situations, or individuals). This paper presents novel evidence for this hypothesis, focusing on a class of Spanish relative clauses that host verbs inflected in the subjunctive. Subjunctive in Romance is standardly taken to be licensed only in a subset of intensional contexts. However, in our relative clauses, subjunctive is exceptionally licensed in extensional contexts. At the same time, the interpretation of these relative clauses still involves modality, a type of modality that targets the goals of the agent of the main event. We argue that the pattern displayed by these relative clauses follows straightforwardly if subjunctive is associated with a modal operator that, like modal indefinites (Alonso-Ovalle and Menéndez-Benito in *Journal of Semantics* 35(1):1–41, 2017), can project its domain from a volitional event. Overall, our proposal supports the event-based analysis of mood (Kratzer in Evidential mood in attitude and speech reports. Talk delivered at the 1st Syncart Workshop, Siena, July 13, 2016; Portner and Rubinstein in *Natural Language Semantics* 28:343–393, 2020) and extends its application beyond attitudinal and modal complements.

## Introduction

A pressing question in research on modality is how modal expressions determine which possibilities they range over. A growing body of work explores the hypothesis that modal domains are projected from particulars (events, situations, or individuals, the ‘modal anchors’) that are made available by the semantic composition. This view, articulated by Kratzer (2013) as the ‘Modal Anchor Hypothesis’, has been recently explored for a number of modal expressions, including modal auxiliaries (Hacquard 2006, 2009, 2010; Arregui 2010), counterfactuals (Arregui 2005, 2007, 2009), modal indefinites (Alonso-Ovalle and Menéndez-Benito 2017), mood (Kratzer 2016; Portner and Rubinstein 2020), and imperfective morphology (Arregui et al. 2014). In this paper, we provide novel evidence for the Modal Anchor Hypothesis by analyzing a class of seemingly exceptional subjunctive relative clauses (RCs) in Spanish, and arguing that their behavior follows straightforwardly if subjunctive is associated with an anchor-sensitive modal operator (Portner and Rubinstein 2020) that, like modal indefinites (Alonso-Ovalle and Menéndez-Benito 2017), can project its domain from a volitional event.

The interpretation of mood in RCs hasn’t received much attention.^1^ Most theories of verbal mood to date focus on the distribution of indicative and subjunctive in the clausal complements of attitude predicates (see Portner 2018 for an overview). For instance, as the Spanish examples in (1) show, desiderative predicates in Romance typically require the verb in their complement clause to bear subjunctive morphology (as seen in (1a)), while doxastic predicates normally select for indicative-marked clauses (as shown in (1b)).^2^ According to a widespread view (what Portner and Rubinstein 2012 call the ‘proto-standard analysis of mood’), the generalization underlying (1) is that subjunctive is licensed only under modal predicates whose semantics is *comparative* and involves a (non-empty) ordering source (see, e.g., Farkas 1992b, Giorgi and Pianesi 1997, Villalta 2006, 2008, among others).^3^ A predicate like ‘want’ intuitively involves comparison, because it focuses on what is best according to the subject’s preferences. On the other hand, a predicate like ‘believe’ describes the subject’s view of reality in its entirety, without designating any part of it as better than another.

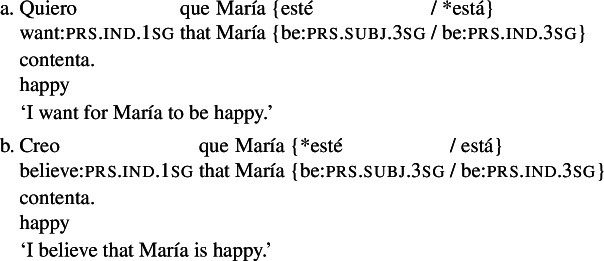

In contrast with clausal complements, Romance RCs under subjunctive licensors exhibit mood alternation: they allow for both subjunctive and indicative, as illustrated for Spanish in (2).

(2)

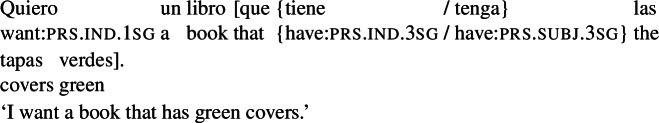

A well-known observation, going back to Quine 1956, is that this mood alternation is linked to the *de dicto*/*de re* distinction. Simplifying slightly, the version of (2) with an indicative RC can only be interpreted as saying that there is a particular actual book that happens to have green covers and that the attitude holder has that book in all worlds conforming to her desires (*de re*). The version with subjunctive in the RC can only convey that in all worlds $w'$ where the attitude holder’s desires are satisfied, she has a book that has green covers in $w'$, with the books potentially varying across the desire worlds (*de dicto*).

The correlation between mood and the *de dicto*/*de re* distinction can be taken to support the (null) hypothesis that mood in RCs has the same licensing conditions as in complement clauses (a hypothesis that has been assumed in various works, e.g., Farkas 1985, Quer 1998, Truckenbrodt 2019; see also Portner 2018 for discussion of this analytical option). On this view, the indicative-marked verb in the RC in (2) would be ruled out in the scope of ‘want’. Assuming that the scope of the RC is determined by the scope of the DP that it belongs to, this would require the whole DP to be interpreted outside of the scope of ‘want’, yielding a specific interpretation. On the other hand, the subjunctive-marked verb would need to stay inside the intensional context to be licensed, which in turn would require the RC (and the whole DP) to remain in the scope of ‘want’, yielding a non-specific interpretation.

This paper focuses on a class of subjunctive RCs that share features of both the indicative and subjunctive RCs in (2). On the one hand, these RCs describe possible states of affairs, like the subjunctive RCs in (2). As we see below, the possibilities introduced by these RCs are those where the goals of an agent are met. Accordingly, we dub them ‘agent-oriented RCs’. On the other hand, like the indicative RCs in (2), agent-oriented RCs are compatible with a specific interpretation of the DP that contains them.

The sentences in (3) provide an illustration. These sentences feature a subjunctive-marked RC (in brackets, with either an object or a subject gap) that is embedded in an object DP. Unlike what we see with subjunctive in cases like (2), the indefinites in (3) receive a specific interpretation: (3a) conveys that a particular actual radio was bought, (3b) that a particular messenger was sent, (3d) that a particular tea was drunk, (3c) that a particular record was bought, and (3e) that a particular book was bought.

(3)

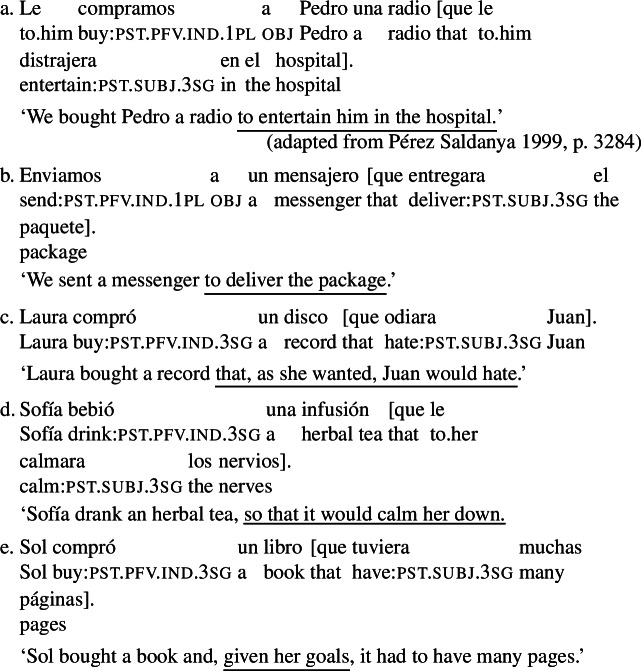

The specific interpretation of the indefinite is not surprising, given that the main verbs in these examples are extensional, and, therefore, license existential import.^4^ At the same time, if subjunctive requires an intensional context, the acceptability of a subjunctive RC in the object position of extensional verbs demands explanation.

The rough translations that we provide above indicate that the interpretation of the RCs in (3) still involves modality, a type of modality that targets the goals of the agent of the event described by the main verb. One way of reconciling the presence of this modality with the specific interpretation of the DP would be to assume that the sentences in (3) involve a covert modal operator taking scope only over the RC. This is a natural assumption, which has been hinted at in previous literature.^5^ But this assumption leads to an equally natural question, which, to our knowledge, has not been previously addressed in depth. The RCs in (3) can only express a particular type of goal-oriented modality: the relevant goals are those that the agent is able to bring about, given the circumstances and what they know (see Sect. 2.1). Given this, an account that assumes a covert modal operator in (3) will need to explain why this operator is restricted to a very particular goal-oriented modal flavor.

We put forward an answer to this question that brings together two independently motivated proposals: (i) that moods introduce modal quantifiers (Kratzer 2016) that, like other modal elements, are anchored to an event and impose selectional restrictions on their anchors (Portner and Rubinstein 2020) and (ii) that goal-oriented modality can be reconstructed from the event argument of a volitional verb (as argued for modal indefinites by Alonso-Ovalle and Menéndez-Benito 2017). We propose that, in examples like (3), the modal quantifier associated with subjunctive is anchored to the VP event and, as a result, it quantifies over worlds compatible with its agent’s goals. In our proposal, both mood selection patterns and the restrictions on modal flavor observed in our RCs are derived by the same mechanism: the selectional restrictions that modals impose on their anchors. This investigation thus paves the way towards a unified theory of verbal mood across categories, a target that, as Portner (2018) notes, research on mood is still far from reaching.

The paper is organized as follows. Section 2 provides a descriptive characterization of the structure and interpretation of agent-oriented RCs in Spanish. Section 3 discusses parallels between agent-oriented RCs and modal indefinites. Section 4 presents background on the view of mood that we build on. Section 5 presents our proposal, applies it to the data set introduced in Sect. 2, and explores the implications of the account for examples that involve multiple instances of subjunctive morphology as well as a wider range of determiners. Section 6 concludes and outlines some questions for further research.

## Characterizing the construction

Agent-oriented RCs are well-documented across Romance: Quer (1998), Laca (2010), and Pérez Saldanya (1999) discuss them in Spanish, Farkas (1985) in Romanian, French, and Italian, and Quer (1998) in Catalan. The construction is subject to cross-linguistic variation: Farkas (1985) discusses differences between Romanian, on the one hand, and French and Italian, on the other. In this paper, we focus exclusively on (Peninsular) Spanish. Unless otherwise noted, our data come from introspective judgments by two of the authors.

In this section, we provide a descriptive characterization of the behavior of Spanish agent-oriented RCs that builds on earlier research but broadens the empirical scope of the discussion. Section 2.1 shows that our RCs can only express a particular type of goal-oriented modality, which targets the goals of the agent of the VP event. Section 2.2 discusses the characterization of agent-oriented RCs in previous work (Farkas 1985; Quer 1998) as ‘purpose relatives’, and Sect. 2.3 shows that this characterization does not cover the full range of data. In Sect. 2.4 we show, building on Quer (1998), that agent-oriented RCs have a restricted distribution. Section 2.5 provides a brief discussion of the choice of tense and aspect morphology in our examples. Finally, Sect. 2.6 offers an overview of the empirical picture, and spells out the research questions to be addressed in the remainder of the paper.

### Modal flavour

As anticipated in Sect. 1, subjunctive RCs in examples like (3) can only express a particular type of goal-oriented modality. First of all, the goals that count are always those of the agent of the main event. Second, those goals have to be within the agent’s reach; they are not mere preferences or desires.

We can illustrate the first property with the example in (4), a variation on (3a). This example is judged false in the scenario in (5), where the radio entertaining Pedro in the hospital does not align with the goals of the agent of the main event (the company).

(4)

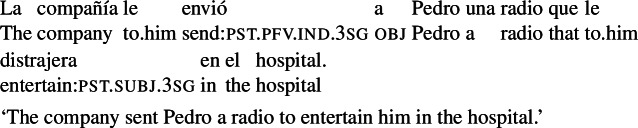

(5) Pedro’s mother wanted him to have a radio that would entertain him in the hospital, and was able to get the company where Pedro works to send him one. The company carried out Pedro’s mother’s request, but had no intentions regarding the outcome (they didn’t care whether the radio entertained Pedro or not).

As for the second property, consider the contrast between (6a) and (6b) in the scenario in (7), inspired by Alonso-Ovalle and Menéndez-Benito (2017). In this scenario, María grabbed a card, and she wanted that card to give her ten points, so the sentence in (6a), featuring ‘want’ in the RC, is true. The sentence in (6b), with the subjunctive *diera* in the RC, says that María took a card, and that her goal was for the card to give her ten points. But for the sentence to be true, achieving this goal should be within María’s reach, in that she should know how to bring it about, and be able to do so. Since the cards are face down, picking a card that would give her ten points is not a goal that María knows how to bring about: (6b) is accordingly false.

(6)

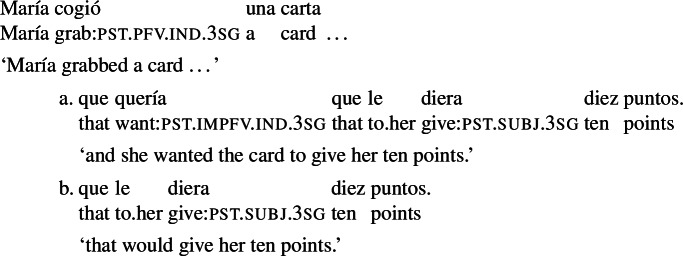

(7) María was playing a card game where all the cards are face down and players pick a card in turns without turning it over. Every type of card gives players a particular number of points. María grabbed a card. She wanted the card to give her ten points.

Given that the goal conveyed by our RCs has to be within the agent’s reach, the sentence in (8) is degraded, as the book turning into a bestseller is presumably not something that Marta can control.

(8)

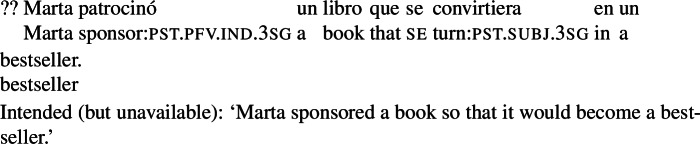

In this connection, we note that examples where the theme of the main verb is the agent of the RC are often ruled out, as seen in (9). This suggests that an event carried out by another agent may not be within reach of the matrix agent in the relevant sense. (We come back to this in Sect. 5.3.1.)

(9)

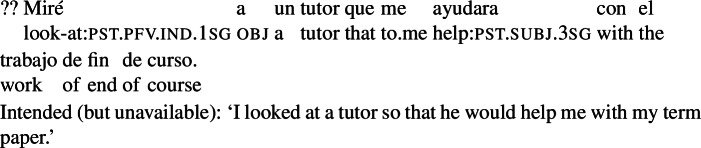

### Previous work: purpose relatives

Previous discussions of agent-oriented RCs (Farkas 1985, Quer 1998, Pérez Saldanya 1999, Laca 2010) focus on RCs like the ones in (3a) (repeated below as (10)), which can be paraphrased with an infinitival purpose clause and are often labelled ‘purpose relatives’ (see Quer 1998, Pérez Saldanya 1999).^6^

(10)

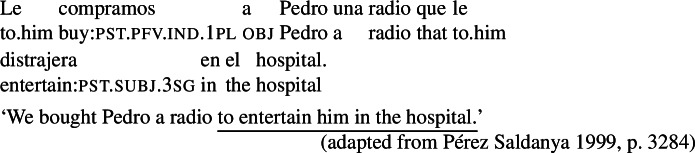

Farkas (1985) argues that in Romanian, French, and Italian, examples like the one above are semantically purpose clauses, and leaves open the question of whether they might be syntactically purpose clauses. To show how this hypothesis might be cast for Spanish, let us briefly present the profile of Spanish finite purpose clauses.

In Spanish, finite purpose clauses are most commonly introduced by the preposition *para* (‘for’) followed by the complementizer *que*, which is homophonous with the relative pronoun *que*. These clauses require subjunctive marking, as seen below.

(11)

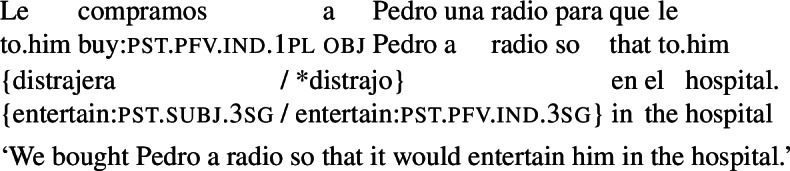

In light of the requirement for subjunctive marking in finite purpose clauses, one might hypothesize that examples like (10) in fact involve purpose adjuncts with the complementizer *que*, where *para* has been omitted for some reason. If that were the case, subjunctive marking in (10) would be expected: whatever is responsible for the licensing of subjunctive with the unambiguous purpose marker *para que* in (11) would also license subjunctive in (10).^7^ As noted above, Farkas (1985) leaves this question open (for Romanian, French, and Italian), but notes in passing that the construction at issue has the hallmarks of an RC.

Quer (1998) presents a number of arguments that convincingly show that (what we are calling) agent-oriented RCs do not in fact involve a purpose adjunct. He focuses on Catalan, but all his arguments carry over to Spanish. In what follows, we illustrate three of them.

#### No preposing

First, agent-oriented RCs cannot be preposed, as shown in (12). They differ in this respect from purpose adjuncts, as seen in (13).

(12)

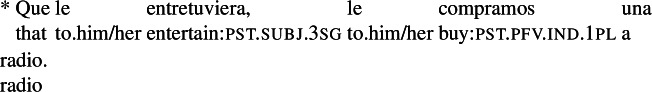

(13)

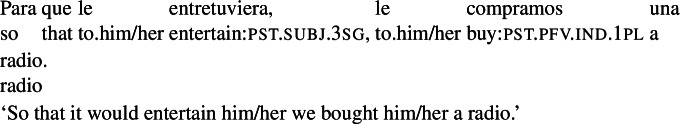

#### Gap

Second, agent-oriented RCs require a gap (as is expected if *que* is a relative pronoun). This is illustrated by the ungrammaticality of (14a), which contrasts with the acceptability of the gapless purpose clause in (14b).

(14)

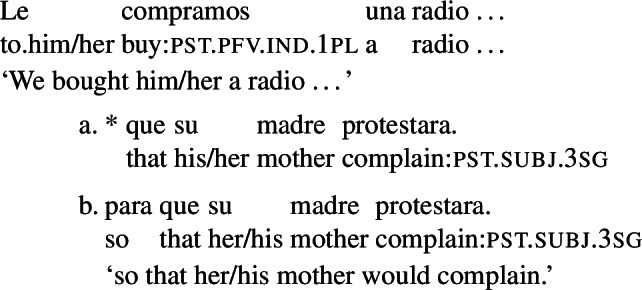

#### Unambiguous relative pronouns

Finally, agent-oriented RCs are available with unambiguous relative pronouns, such as *cuyo* (‘whose’) in (15).

(15)

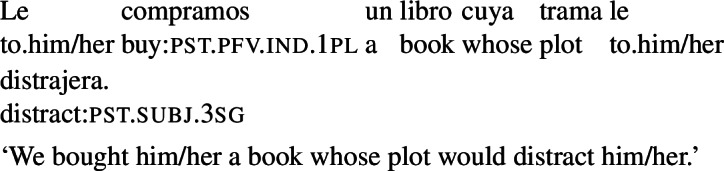

Quer’s arguments clearly show that agent-oriented RCs do not involve a purpose adjunct syntactically. In the next section, we show that, contrary to what has been assumed in previous literature, these RCs are not always amenable to a purpose paraphrase either, as they do not require prospective temporal orientation.

### Not only prospective orientation

Previous literature on agent-oriented RCs focuses on examples that can be paraphrased with a purpose infinitival clause (e.g., ‘we bought him a radio to entertain him.’) To these, we can add examples like (3d), which can be paraphrased in English by means of a finite purpose clause, introduced by *so that* (‘Sofía drank an herbal tea so that it would calm her down.’) All these examples share the prospective orientation of a purpose interpretation: they introduce a situation that the agent of the main verb intends to bring about.

Prospective temporal orientation is, however, not a necessary ingredient of the construction. Examples like (3e), repeated below as (16), lack this sense of bringing about a future eventuality.^8^

(16)

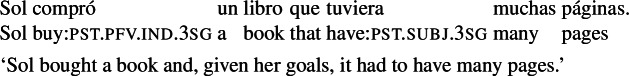

The sentence is not interpreted as saying that the book’s having many pages was a goal that the agent intended to bring about. Rather, the RC in this example mentions the criterion that the agent was guided by in selecting the book she bought: (16) conveys that Sol would have only bought a book that had many pages (at purchase time). Note that (16) would be considered false in a situation where Sol happened to buy a book with many pages but *not for that reason*. For instance, (16) is judged true in the scenario in (17a) but false in (17b).

(17)
  - ***Millenium*** **I.** Sol’s friend Marta was taking a long train ride and Sol decided to buy her a long book that would keep her entertained throughout the trip. She went to the bookstore and bought a book that had that property. It happened to be the first book in the Millenium trilogy.
  - ***Millenium*** **II.** Sol decided to buy her friend Marta a Swedish thriller, since she loves the genre. She found on the shelf the first book in the Millenium trilogy, so she bought that one. (But she would have preferred to buy a shorter book, as Marta easily gets tired of reading.)

Quer’s arguments can be replicated for examples like (16), establishing that they involve true RCs (not verbal modifiers). In particular, *que tuviera muchas páginas* cannot be preposed (see (18)) and it includes a gap (see (19)). Additionally, it is possible to construct non-prospective examples with the unambiguous relative pronoun *cuyo* (witness (20)).

(18)

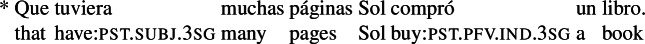

(19)

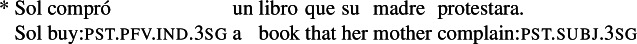

(20)

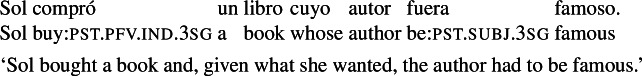

Agent-oriented RCs can also be backshifted with respect to the running time of the main event. Although Pérez Saldanya (1999) remarks that ‘purpose relatives’ disallow past orientation, backshifted agent-oriented relatives *are* possible if it is plausible that the (past) property expressed by the RC guided the agent in making her choice, as in (21) below.

(21)

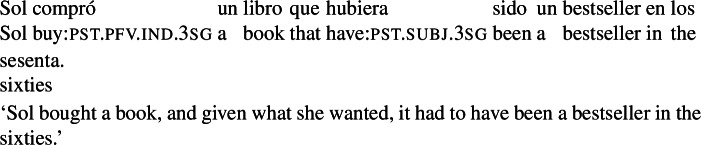

Non-prospective agent-oriented RCs differ from the RCs discussed in Sect. 2.2 in that they convey that the actual object has the property denoted by the RC: (16) signals that the book bought actually has many pages, and (21) that it was actually a bestseller in the sixties. In contrast, our radio example (3a) can be true even if the radio ended up not entertaining Pedro. Despite these differences, we argue in Sect. 5 that agent-oriented RCs, prospective or not, should receive a unified analysis. Contrasts between examples like (3a) and examples like (3e)/(16) are traced back to their temporal orientation, which in turn is partially determined by the combination of tense/aspect morphology and aspectual class.

As the reader will have noticed, all our agent-oriented RCs so far involve past subjunctive^9^ (for reasons behind this choice, see Sect. 2.5.) But just like in the case of subjunctive conditionals (see, e.g., Iatridou 2000, and much follow-up work) this past morphology is ‘fake’ in that it does not signal anteriority. Our radio example, for instance, is compatible with situations in which the radio was meant to entertain Pedro before the time of utterance, and with situations where the entertaining was supposed to take place after the time of utterance.^10^ This is illustrated in (22), where the two possibilities are brought out by temporal modifiers.

(22)

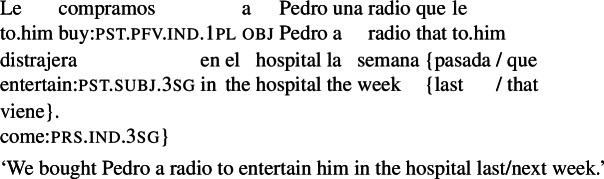

When the RC verb involves one layer of past morphology, the temporal orientation of the RC partially correlates with aspectual class, like in the case of subjunctive conditionals and the complement of modal auxiliaries.^11^ Eventive predicates in the RC force a prospective interpretation, as in (3a). Individual-level predicates require a simultaneous interpretation, as in (16). Stage-level statives in principle allow for both possibilities, as the example in (23) shows. To get a backshifted interpretation, we need an additional layer of past, as in (21).

(23)

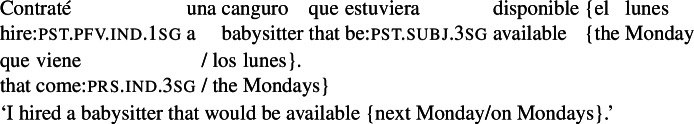

### Restricted distribution

Quer (1998) noted that (what he labelled) ‘purpose relatives’ have a restricted distribution: they are only possible when the main verb is volitional. The following examples show that this restriction applies to agent-oriented RCs regardless of their temporal orientation. With non-volitional verbs like *descubrir* (‘discover’), an object containing a subjunctive RC is degraded, whereas an indicative RC is fully acceptable, whether the RC is prospective (24) or not (25).

(24)

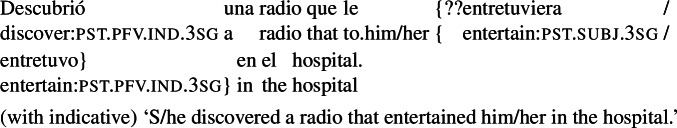

(25)

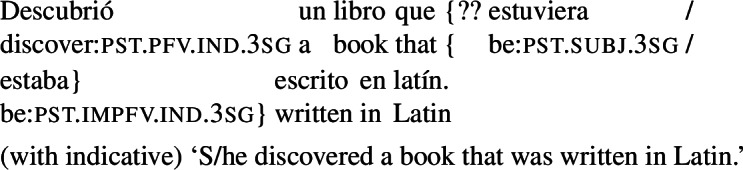

The volitionality requirement can be further illustrated by showing that adding a modifier like *sin darme cuenta* (‘without realizing’) to otherwise felicitous examples makes them degraded (as noted by Farkas (1985) and Quer (1998) for ‘purpose relatives’). This is shown in (26) below.

(26)

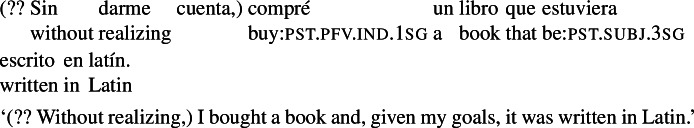

A further and hitherto unnoticed restriction is that agent-oriented RCs are not possible in the *subject position* of (active) volitional verbs. While the sentence in (27) (a variation on our radio example) is fully grammatical, an attempt to place a subjunctive RC in the subject position of *visitó* (‘visited’) results in ungrammaticality, as shown in (28). An indicative RC in this configuration is unproblematic.

(27)

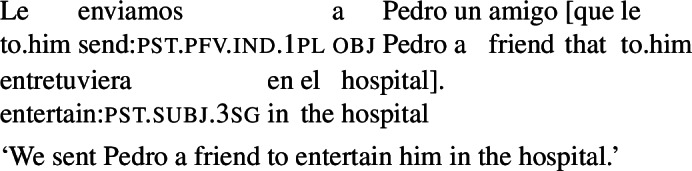

(28)

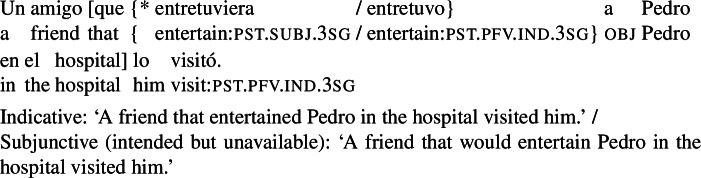

The same pattern can be illustrated with non-prospective RCs: the version of (29) with a subjunctive RC is ungrammatical, whereas its indicative counterpart is perfectly fine.

(29)

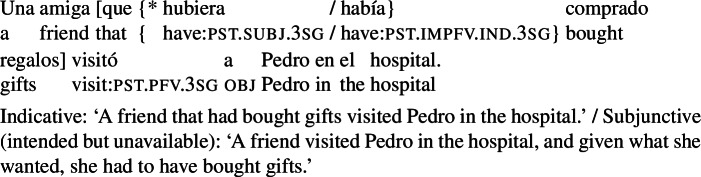

The factor that rules out subjunctive in (28) or (29) is that the RC is part of the external argument of the verb. First, note that this restriction is independent of word order: placing an agent-oriented RC in a postverbal subject does not make it acceptable. This is shown in (30) for the VOS version of (28), which is ungrammatical with a subjunctive RC, but not with an indicative RC.^12^

(30)

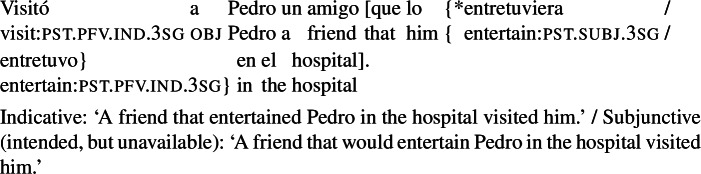

Second, agent-oriented RCs are acceptable in the subject position of *passive* verbs. While stilted, (31) is grammatical. This shows that what blocks subjunctive in (28) is agentivity, not syntactic subjecthood.

(31)

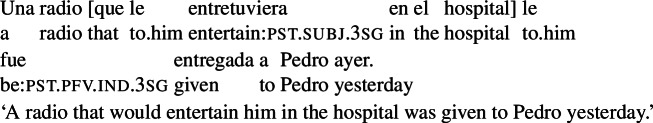

Finally, agent-oriented RCs are possible not only in the direct object position of volitional verbs, but also in other constituents within the VP layer. The example in (32) illustrates this for an indirect object.

(32)

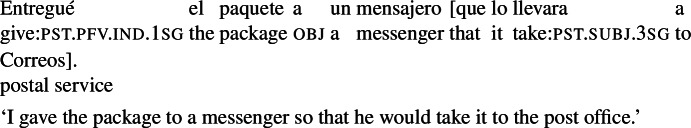

### A few words about tense and aspect

Before wrapping up this section, we would like to comment on the tense and aspect combinations we use in our examples. In all the examples so far, the main verb bears perfective morphology. This is not a necessary ingredient of the construction, though. The example in (33), for instance, features an agent-oriented RC in a sentence with an imperfective main verb.

(33)

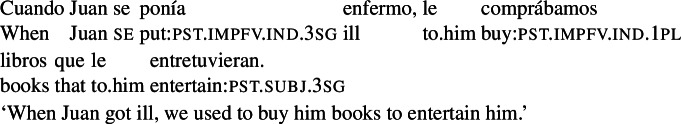

Our focus on perfective main verbs is a methodological choice, as most other tense and aspect combinations on the main verb could be argued to introduce an independent layer of modality. For instance, imperfective-marked (or present tense) verbs can have habitual (as in (33)), progressive, or futurate readings, all of which are amenable to a modal account (see Arregui et al. 2014 for recent discussion). Similarly, future morphology is commonly taken to contribute a modal component. The choice of matrix past (*vs.* future) and perfective (*vs.* imperfective) morphology ensures that the RC occurs in a truly extensional environment. This allows us to set up the puzzle more transparently.^13^

In addition, as noted in Sect. 2.3, in all our target examples the RC verb occurs in the past subjunctive form. This is not a requirement of the construction either; the sentence in (34) shows that it is possible to construct agent-oriented RCs with a present subjunctive verb.

(34)

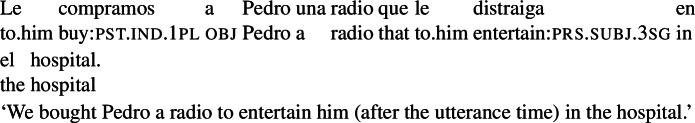

It is again our methodological choice to focus on examples in which the RC verb is in the past subjunctive. Present subjunctive RCs require non-past temporal reference: (34) is not felicitous if the radio was supposed to entertain Pedro at a past time. Accordingly, it is not possible to construct examples where a present subjunctive RC temporally overlaps the event introduced by the past main verb. As shown in (35), a version of our book example (16) with a present RC is pragmatically odd, as it conveys an implausible connection between the buying of the book and the book’s future length.

(35)

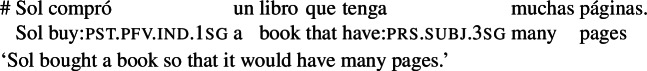

The use of a past subjunctive verb gives us more flexibility with respect to the temporal interpretation of the RC. This has allowed us to include in our data set RCs that do not have a prospective interpretation (Sect. 2.3), thus broadening the empirical scope of the discussion.

### Interim summary and research questions

Let us take stock. We have seen that agent-oriented RCs are true relative clauses (not adverbial modifiers of the verb). Their interpretation is relativized to an agent’s goals, but, unlike purpose clauses, they do not require prospective temporal orientation. The distribution of agent-oriented RCs is restricted in that they are disallowed with non-volitional verbs, and in the external argument position of volitional verbs.

The pattern displayed by agent-oriented RCs raises the following questions:

1. **The source of modality:** what introduces goal-oriented modality in our RCs? The answer to this question has to be consistent with the fact that our target examples involve existential import, as seen in Sect. 1.
2. **The type of modality:** what fixes the particular modal flavor?
3. **The restricted distribution:** what explains the restricted distribution of agent-oriented RCs?

Quer (1998), when discussing (what he called) ‘purpose relatives’ in extensional contexts, argued that the modal component in these examples is recovered from the volitional agent. On his view, the presence of the agent allows us to retrieve a set of worlds (a *model* in his terms) that represents the intentions of the agent.

Taking Quer’s ideas as a starting point, our aim in the rest of the paper is to provide an explicit account of how the modality in agent-oriented RCs is introduced in the semantic composition. To achieve this, we marry two recent lines of research: (i) work on modal indefinites (Alonso-Ovalle and Menéndez-Benito 2017), and (ii) an approach to verbal mood in which mood introduces a modal operator anchored to an event (Portner and Rubinstein 2020). We claim that, in our examples, this modal projects its domain from a volitional event, following the recipe motivated for modal indefinites by Alonso-Ovalle and Menéndez-Benito (2017). The following two sections set the stage by discussing the parallisms betwen agent-oriented RCs and agent-oriented modal indefinites (Sect. 3), providing an overview of the approach to verbal mood that we adopt (Sect. 4), and establishing the connection between the two domains.

## Random choice indefinites and modal projection

Across languages, we find existential determiners that express modality in the absence of other modal expressions (Haspelmath 1997). A sub-class of these are ‘random choice indefinites’: indefinites that express agent-oriented modality, indicating that an agent made an indiscriminate choice.^14^ The Spanish indefinite *uno cualquiera* (henceforth UC) belongs to this category. The example in (36), for instance, conveys that Juan grabbed a card and additionally signals (roughly) that grabbing any other card would have been *compatible with Juan’s goals*.

(36)

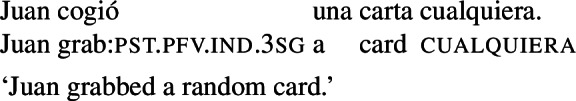

The behavior of UC exhibits striking parallelisms with our agent-oriented RCs. The discussion below illustrates these parallelisms, closely following the characterization of UC by Alonso-Ovalle and Menéndez-Benito (2017).

First of all, the random choice interpretation associated with (36) has a restricted distribution. To show this, let us start by noting that examples like (36) have, apart from the random choice interpretation paraphrased above, an additional ‘evaluative’ reading (that Juan bought a book that the speaker considers unremarkable). While the evaluative interpretation is always available, the random choice interpretation is disallowed with non-volitional verbs and in the subject position of active volitional verbs, the very same configurations that disallow our agent-oriented RCs (Sect. 2.4). The minimal pair in (37) illustrates the volitionality restriction: (37a), with a volitional agent, has both the random choice and evaluative interpretations, but (37b) only has the evaluative reading (as the yeast lacks intentions). The non-subject restriction is illustrated in (38). This example, where *un estudiante cualquiera* is in the subject position of an active verb, can only convey that an unremarkable student spoke. The example in (39) shows that the random choice reading is possible in the subject position of passive verbs (while somewhat stilted, (39) is clearly grammatical).

(37)

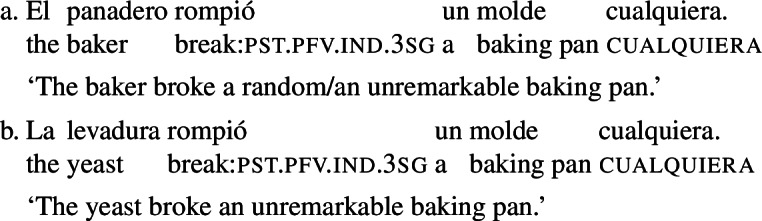

(38)

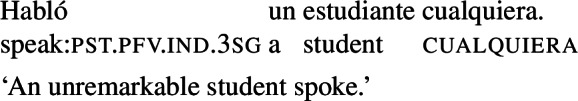

(39)

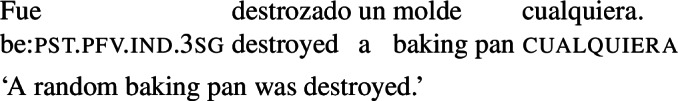

Second, the type of agent-oriented modality conveyed by UC targets what Alonso-Ovalle and Menéndez-Benito (2017) call ‘action goals’: roughly, these are goals tightly connected to the agent’s decision to act, in the sense that they are outcomes that the agent is able to bring about, *given the circumstances and the agent’s epistemic state*. Alonso-Ovalle and Menéndez-Benito (2017) illustrate this with the following contrast: while (36) is judged as false in the scenario in (40b), it is true in the scenario in (40a). In both scenarios, Juan wanted to take the ace. Why is then (36) true in (40a)? It looks like taking other cards was *not* compatible with what Juan wanted to achieve. The reason is that, even though Juan wanted to take the ace, he could not decide to do so, because he lacked the necessary information (the cards were face down). Given what he knew, all he could decide was to take a card, any card, and hope for the best. It is in this sense that taking any card was compatible with Juan’s action goals. There are worlds compatible with his action goals where he takes the ace of spades and worlds compatible with his goals where he takes the queen of hearts. That’s not the case in the scenario in (40b). In that scenario, Juan wanted to take the ace, and decided to do so. His action goals are not compatible with taking any card (they exclude taking the queen), so (36) is accordingly false.

(40)
  - There were two face-down cards in front of Juan. Juan knew that one was the queen of hearts and the other the ace of spades. He wanted to take the ace but didn’t know which card was which. He took a card at random.
  - There were several face-up cards in front of Juan. Juan wanted to take the ace of spades and he did so.

This type of goal-oriented modality is closely related to the modality conveyed by our agent-oriented RCs. As discussed in Sect. 2.1, the example in (41) is false in a situation where María wanted to take a card that would give her ten points but the cards in front of her were face-down. To preview our proposal, we will say that (41) conveys that the card that María took gives her ten points in all worlds compatible with her action goals. But, just like in (40a), all that María could decide in a cards face-down scenario was to take a card, any card (and hope that this card would give her 10 points). Given this, there will be worlds compatible with her action goals where the card that she took doesn’t give her 10 points.^15^

(41)

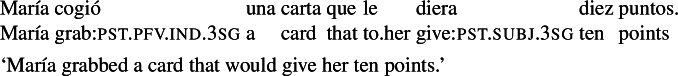

Under the analysis proposed by Alonso-Ovalle and Menéndez-Benito (2017), which we discuss here in simplified terms, the modal domain of UC consists of the set of worlds compatible with the goals associated with the agent’s decision to act. The sentence in (36) accordingly has the truth conditions in (42).^16^

(42) True in *w* iff
  - there is a past event *e* of Juan taking a card *x* in *w* and
  - for every relevant card *y* there is a world $w'$ compatible with Juan’s action goals in *w* where there is an event $e'$ of Juan taking *y*.

How does UC access the agent’s goals? Abstracting away from the compositional details, the gist of Alonso-Ovalle and Menéndez-Benito’s (2017) proposal is as follows: UC introduces a modal component that can only be anchored to events that determine goals. Volitional events satisfy such a condition. From a volitional event *e*, UC will be able to retrieve a set of possible worlds: those where the circumstances surrounding *e* obtain and that are best with respect to the goals of the agent of *e*.

The parallelism in interpretation and distribution between UC and our agent-oriented RCs^17^ would follow if, like UC, subjunctive mood were analyzed as a modal element whose interpretation is relative to an event. This analysis has in fact been proposed in the recent literature on mood. We turn to its components next.

## Mood as modality

Research on mood, specifically in Romance languages, has focused on so-called *mood selection*, where a particular choice of mood marking is required in the complements of different propositional attitude verbs (see Farkas 1992b, 2003, Giannakidou 1997, Giorgi and Pianesi 1997, Portner 1997, 2018, Quer 1998, 2020, Schlenker 2005, Villalta 2006, 2008, Smirnova 2011, Anand and Hacquard 2013, Giannakidou and Mari 2021, among others). Mood morphemes have usually been taken to depend on a licensing modal operator: ‘want’ or ‘believe’ in examples like (1), following a Hintikkan analysis (Hintikka 1969). As noted in Sect. 1, mood selection has been argued to turn on whether or not the propositional attitude is comparative. While implementations vary, the semantic contribution of the mood morphemes tend to be analyzed as contributing a restriction, typically in the form of a presupposition on the domain of quantification of the licensing modal.

In recent years, this general view about mood semantics has been shifting, in a way that we argue opens up new possibilities for the analysis of mood in RCs. On the one hand, the Hintikkan analysis has been challenged by the proposal that modality in attitude ascriptions originates from outside the attitude verb (Kratzer 2006, Moulton 2009). On this view, attitude verbs may denote predicates of events as illustrated in (43).^18^

(43)

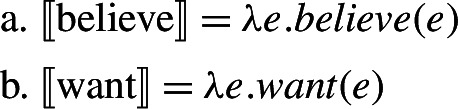

Building on Hacquard (2006, 2010), Kratzer also assumes that modal operators project their domains (that is, identify the set of accessible worlds they range over) from particulars such as events or individuals, the ‘modal anchors’ (for discussion of this hypothesis, see Kratzer 2013). This, together with the assumption that attitude verbs are not themselves modal, provides a straightforward explanation of *harmonic modality* (Lyons 1977). In (44), for example, *should* is said to be harmonic with *advised*, in the sense that the sentence contributes only one layer of modality. It conveys that in all worlds consistent with the advice, we establish (not: we should establish) an emergency fund (see Kratzer 2016).

(44)

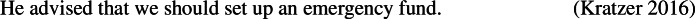

Kratzer (2016) draws a novel parallel between modals and moods, and argues (using the German reportative subjunctive as a case study) that moods, rather than being *selected* by attitude verbs, are responsible for *creating* the modal semantics associated with attitude ascriptions.

Focusing back on Romance, Portner and Rubinstein (2020) propose a theory of mood selection in Spanish and French in which mood morphology introduces modal quantification, attitude verbs are predicates of events, and the latter provide the anchor for the former. Moods are assigned the schematic denotation in (45a), which relates an attitude event to the embedded proposition, much like a thematic role relates the event to its (experiencer) participant (45b).

(45)
  - 〚mood〛 = *λpλe*.*Necessity*(*p*,*e*) (to be elaborated)
  - 〚exp〛 = *λxλe*.*Experiencer*(*x*,*e*)

On this proposal, the modal flavour of mood is determined by the attitude event anchor. Portner and Rubinstein assume that every attitude event *e* is associated (lexically) with modal backgrounds, which they refer to as the *content* of *e*. These backgrounds are used to set up the quantificational domain of mood.^19^ In these terms, the content of a wanting event, for example, is a pair of backgrounds 〈*doxastic* + ,*bouletic*〉 (i.e., a certain doxastic modal base and a bouletic ordering source),^20^ whereas the content of a believing event is a single background (a doxastic modal base).^21^

On Portner and Rubinstein’s (2020) account, presented in simplified form in (46), the difference between the indicative and subjunctive in Romance resides in their quantificational force. Indicative is a strong necessity modal, which quantifies over all worlds determined by a modal base. It is defined just in case its eventuality anchor *e* is associated with a single modal background (see (46a)). Subjunctive, on the other hand, is only defined for eventualities that are associated with two backgrounds, as in (46b). It is a weaker necessity modal, which quantifies over only the accessible worlds (selected by a modal base) which rank best with respect to an ordering source (see also Matthewson 2010).^22^ We assume that both events and worlds are of the same type, *s*.

(46)

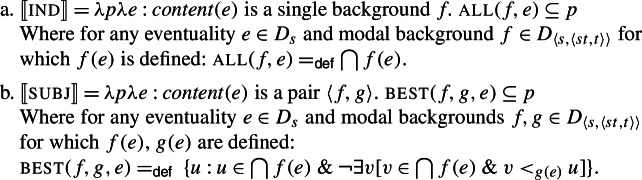

The function in (46a) maps an event *e* and proposition *p* to truth just in case the content of *e* is a single modal background, and all worlds consistent with this background at *e* (for instance ⋂(*doxastic*(*e*)) for an event *e* for which *content*(*e*)=*doxastic*) are worlds where *p* is true. The function in (46b) maps an event *e* and proposition *p* to truth just in case the content of *e* is a pair of modal backgrounds and the modal-base worlds which are best given the ordering source associated with *e* are worlds where *p* is true.^23^

On this proposal, patterns of mood selection follow directly from the semantic composition. Indicative can only combine with events that supply just one modal background (e.g., believing events) whereas subjunctive requires its event argument to provide two modal backgrounds (as, e.g., wanting events do). If the selectional properties of mood are satisfied, composition will be succesful. We illustrate this for (1b) in (47), with the prototypical indicative selector *creer* (‘believe’). For ease of presentation, we ignore the contribution of tense and use English translations instead of the Spanish original.

(47)

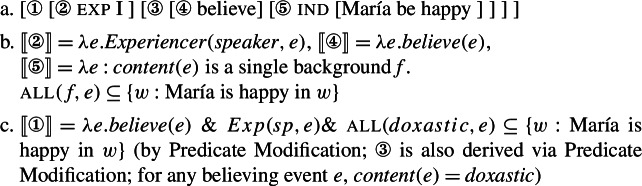

Conversely, composition will fail due to presupposition failure if the attitude event does not provide enough modal backgrounds (for example if we combine ‘believe’ + subj) or too many of them (in the case of ‘want’ + ind).^24^

The event-based approach to mood is a recent development. It was designed to account for one type of mood marking, namely selected mood, raising the question of its applicability to additional enviroments where mood inflection plays a role, e.g., in the scope of negation or in RCs. Two immediate challenges present themselves in extending the analysis to the subjunctive RCs characterized in Sect. 2. First, what provides an anchor for the modality introduced by mood in the absence of an attitude event? Second, how are multiple occurrences of mood interpreted, in sentences that contain both a subjunctive RC and a subjunctive-selecting attitude?

We focus first on the anchor question. On the view of mood that Portner and Rubinstein put forward, we expect mood to be grammatical, even in the absence of an attitude event, so long as it can combine with an event argument with the right type of content. In Sect. 5 we claim that subjunctive may find its anchor in the event argument of the main verb, just like the agent-oriented modal indefinites analyzed by Alonso-Ovalle and Menéndez-Benito (2017). Section 5.4 takes up the challenge of interpreting multiple moods.

## Proposal

The parallelism in interpretation and distribution between agent-oriented RCs and the random choice reading of UC is striking. As anticipated, we argue that this parallelism obtains because the random choice reading of UC and the modal quantifier associated with subjunctive in our RCs project modality from the same modal anchor: the event argument of the main verb.

Section 5.1 spells out this idea, thereby addressing two of the research questions outlined in Sect. 2.6: a modal associated with subjunctive morphology is the *source* of modality in our examples; the particular *modal flavour* comes about via event-anchoring. Section 5.2 focuses on our third research question: what explains the distributional restrictions of agent-oriented RCs? In Sect. 5.3, we take a closer look at the nature of the accessible worlds invoked by agent-oriented RCs. Section 5.4 discusses examples featuring multiple instances of subjunctive morphology. We propose that these examples involve modal concord, which, following Zeijlstra (2007), we analyze as an agreement phenomenon. Finally, Sect. 5.5 offers some discussion of the types of determiners that can appear in our RCs.

### Anchoring agent-oriented RCs

Recall that, on Portner and Rubinstein’s account, the Spanish subjunctive introduces a modal quantifier that takes an event argument whose content determines a modal base and an ordering source (as in (48)). And given Alonso-Ovalle and Menéndez-Benito’s (2017) account of UC, volitional events can be argued to meet this condition. Casting Alonso-Ovalle and Menéndez-Benito’s proposal in the framework introduced in Sect. 4, we can think of the content of a volitional event as a pair of modal backgrounds, specifically a circumstantial modal base and a teleological ordering source, as in (49). We claim that this is the source of the goal-oriented modality in agent-oriented RCs.

(48) 〚subj〛 = *λpλe*:*content*(*e*) is a pair 〈*f*,*g*〉. best(*f*,*g*,*e*)⊆*p*

(49) If *e* is a volitional event, then *content*(*e*) is the pair 〈*circumstantial*,*goal*〉 where
  - *circumstantial(e)*: circumstances surrounding *e*
  - *goal(e)*: goals associated with the agent of *e*

We assume that the sentence in (50) is associated with the LF in (51), where world and event arguments of predicates and their binders are syntactically represented (Hacquard 2006) and agents are introduced by a separate functional head (Kratzer 1996) (we do not show this at the level of the RC, only for simplicity.). Furthermore, we assume that the event argument of the modal quantifier introduced by mood in RCs is also syntactically represented and obligatorily co-bound with another event in the structure (Alonso-Ovalle and Menéndez-Benito (2017) make the same assumptions for the event argument of UC). In (51), the event argument of subjunctive can only be co-bound with the event argument of the main verb, but as we will see below, co-binding with a higher event is in principle possible when the RC is further embedded.

(50)

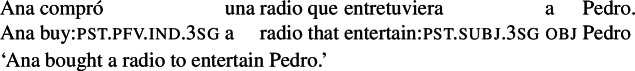

(51)

Given (48) and (49), the semantic composition will yield the truth conditions in (52) for the LF in (51): that there is an event *e* of Ana buying Pedro a radio and in all circumstantially accessible worlds that are best given the goals associated with *e*, that radio entertains Pedro.

(52)

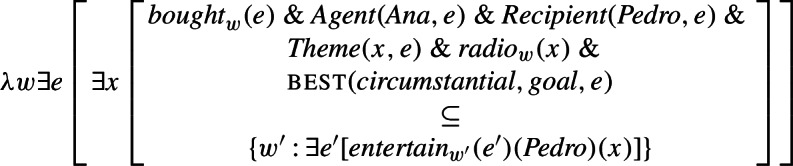

These truth conditions deliver the two seemingly conflicting properties of agent-oriented RCs that we noted in Sect. 1: that they license existential import (a result of the fact that the modal operator scopes only over the RC) and that they express goal-oriented modality.

A couple of remarks about our syntactic assumptions are in order. First, note that we are assuming, as Portner and Rubinstein (2020) do, that subj sits at the left periphery of the RC. This is in principle compatible with two analytical options. The first one is that subjunctive morphology originates in the TP and moves to the C position at LF in order to scope over the proposition denoted by the TP (Portner and Rubinstein 2020, p. 385). The second is that subjunctive morphology on the verb reflects the presence of a quantifier base-generated in C. For now, we do not decide between the two options, as the data we have considered so far do not distinguish between them. In Sect. 5.4, we discuss examples featuring several instances of subjunctive morphology and propose an analysis of these examples that adopts the second option above.

Second, we assume that the event argument of mood can be co-bound with any event in the structure that meets the relevant selectional restrictions. We want our system to be flexible in this way, as event anchoring does not need to be local: in the example in (50), the only possible anchor for subj is the VP event, but we can find examples where mood is anchored to an event in a higher clause. For instance, in (53) the modal quantifier in the RC in brackets ranges over the worlds where the architect’s promises are met. To derive this interpretation, we need to assume that the event argument is identified with the promising event.^25^

^,^ 26

(53)

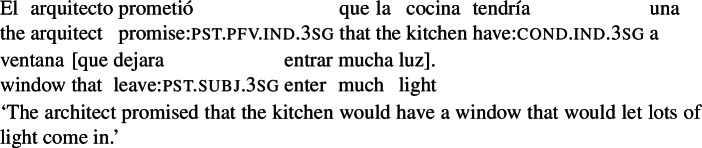

Let us now briefly come back to the differences between examples like (50) and examples like (3e), repeated below as (54). As noted in Sect. 2.3, the eventive predicate in the RC in (50) forces a prospective interpretation (the event of entertaining has to temporally follow the event of buying), while the individual-level predicate in (54) determines a simultaneous interpretation.^27^

(54)

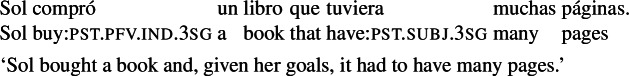

As discussed in Sect. 2, (50) relates to a goal that the agent intended to bring about, while (54) expresses the criterion that the agent was guided by when selecting the object she acted upon. We contend that this interpretative contrast follows simply from the fact that agents cannot intend to bring about states of affairs that are independently determined and that our analysis nevertheless derives the correct truth conditions when this is the case.

On our account, (54) is predicted to be true only if the agent bought a book and, in all worlds that best conform to her goals, the book had many pages at the time of buying. This will give rise to the intuition that the goal would only be satisfied by buying a book with many pages. The same intuition holds for backshifted agent-oriented RCs (recall (21), which conveys that the agent’s goal was satisfied by buying a book that was a bestseller in the sixties). In Sect. 5.3.2, we show that this intuition crucially relies on the modal base being *diverse* with respect to the RC. Additionally, Sect. 5.3.2 shows that the intuition that the property expresed by the RC holds of the actual book follows once we take into account the types of goals at play in agent-oriented RCs (see Sect. 5.3.1).

If RCs like the ones in (54) and (21) are understood as expressing the criterion that guided the agent because they denote properties that are independently determined, we might expect future-oriented RCs that introduce (independently) scheduled events to give rise to the same intuition. This expectation is met. The example in (55) intuitively conveys that the agent selected the actor based on the fact that he was scheduled to perform.

(55)

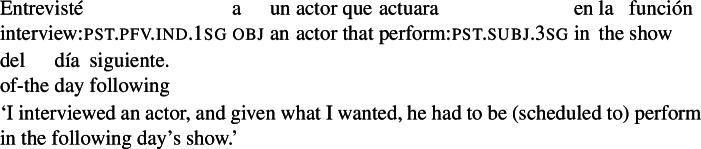

### Distribution

We turn now to the distributional restrictions that agent-oriented RCs share with (the random choice reading of) UC, i.e., their unavailability (i) in the external argument position and (ii) with non-volitional verbs.

#### The external argument restriction

We propose that agent-oriented RCs are ruled out in the subject position of active verbs because subjunctive cannot access the verb’s event argument in the resulting configuration. The subjects we are dealing with are quantificational, hence of a type that prevents them from composing directly with the Agent thematic role. While we remain agnostic about the precise position of quantificational subjects of active verbs at LF (both preverbal and postverbal), we assume that they are higher than the locus of the existential closure of the event argument.^28^ This is illustrated in the schematic structure in (57), corresponding to the sentence in (56) (the subjunctive variant of (28)). In this configuration, the anchor of subjunctive cannot be co-bound with the verb’s event argument, because the existential quantifier that closes off the event argument is lower than the subject.

(56)

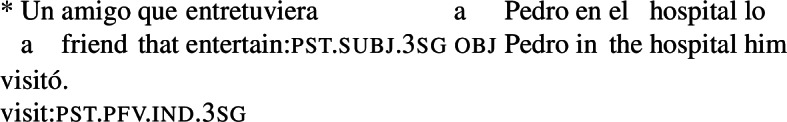

(57)

In contrast, if we assume that passive subjects remain below the locus of existential closure at LF, we expect that the modal anchor of a subjunctive RC within them could be co-bound with the event argument of the main verb. This is shown for the sentence in (31), repeated in (58) below, assuming the schematic LF in (59).^29^

(58)

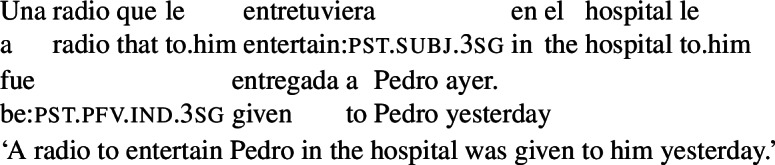

(59)

#### The volitionality restriction

We turn now to the unavailability of agent-oriented RCs with non-volitional verbal predicates, such as (60), repeated from (24).

(60)

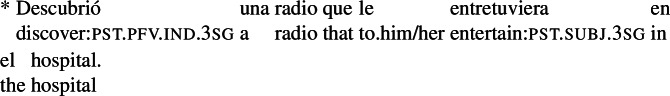

What we have said so far predicts that the RC in (60) should not get a goal-oriented interpretation: since non-volitional events do not have agents, they do not evoke agent goals and cannot determine a goal-oriented ordering source.

But we are still left with the question of why we cannot retrieve a different kind of ordering source from non-volitional events. For instance, one might imagine that these events supply a likelihood or stereotypical ordering source like the one that subjunctive interacts with in examples like (61).

(61)

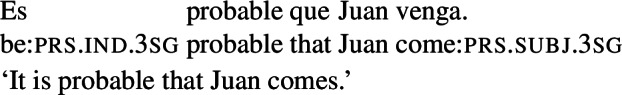

We do not have a full-fledged answer to this question, but we would like to make the following suggestion. A stereotypical ordering source characterizes what the most likely course of events is, given what has happened up till now and general facts about the world. Reconstructing this kind of ordering source would require ‘looking beyond’ the properties of the event that we take as the modal anchor. We suggest that this kind of zooming out is blocked by the grammar: when we determine a domain of possibilities from an event, we can only do so by looking at the intrinsic properties of the event, such as what kind of event it is, and who its participants are.

### The nature of the accessible worlds

In this section, we discuss in greater detail the flavour of the modality expressed by agent-oriented RCs. In Sect. 5.1, we claimed that subjunctive in these RCs is interpreted with respect to a circumstantial modal base and a teleological ordering source, retrieved from a volitional event as in (62).

(62) If *e* is a volitional event, then *content*(*e*) is the pair 〈*circumstantial*,*goal*〉 where
  - *circumstantial*(*e*): circumstances surrounding *e*
  - *goal*(*e*): goals associated with the agent of *e*

But what goals count? What exactly are the circumstances surrounding *e*? In what follows, we address these questions in turn. Section 5.3.1 reviews the type of goals that go into the teleological ordering source, elaborating on the discussion in Sects. 2.1 and 3. Section 5.3.2 returns to our book example, and shows that this kind of example motivates a more fine-grained characterization of the circumstantial modal base in agent-oriented RCs.

#### The teleological ordering source: the agent’s decision

In Sect. 2.1, we noted that the goals relevant for the interpretation of agent-oriented RCs are goals that the agent is able to bring about. In Sect. 3, we made a parallelism with the goals that UC is sensitive to: the ‘action goals’ associated with the agent’s *decision to act* (Alonso-Ovalle and Menéndez-Benito 2017). Adapting their discussion to our framework and simplifying somewhat,^30^ for a proposition *p* to be an ‘action goal’ for agent *a*, bringing *p* about has to be within *a*’s control: *a* has to know how to bring about *p*, and be able to do so, given the circumstances.

As anticipated in Sect. 3, we contend that goal-oriented RCs are also (only) sensitive to action goals. This is why the example in (6a), repeated below as (63), is false in a scenario where María wanted to take a 10-point card, but the cards are face-down (see Sect. 2.1). In such a scenario, taking a card can be an action goal of María’s, but taking a card that would give her 10 points is not. Given this, the worlds that are best with respect to her action goals will vary with respect to whether the card she took gives her 10 points or not.

(63)

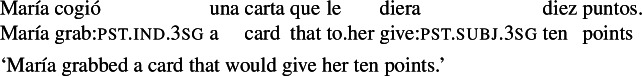

The sentence in (8), repeated as (64), will be systematically false given common knowledge: turning the book into a bestseller is, under normal circumstances, not a possible action goal for Marta. This, we contend, accounts for its oddity.

(64)

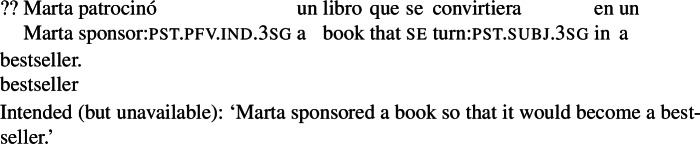

What about the examples in (65)? We suggest that a goal can only be an action goal for an agent *a* if *a* is able to bring it about without resorting to the cooperation or authority of other agents.^31^ On this view, (65) is degraded when the main verb is ‘look at’ because the agent of the looking event cannot bring about the tutor’s help without the tutor’s cooperation. In contrast, the variation with ‘hire’, which involves a contract between the two agents, is perfectly fine.^32^

(65)

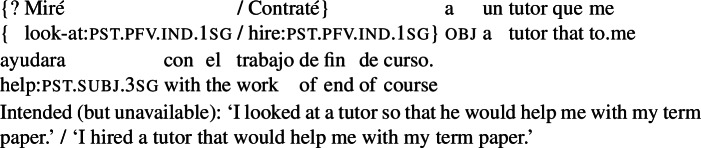

As an anonymous reviewer notes, it is possible to rescue cases where the requirement for agent control is not satisfied, by switching to an interpretation in which the RC event is (independently) planned. For instance, the example in (66) cannot mean that the agent looked at a tutor *so that* he would present the following Monday. It is grammatical, however, and means that the tutor in question planned to present, and that prompted the agent to look at him.

(66)

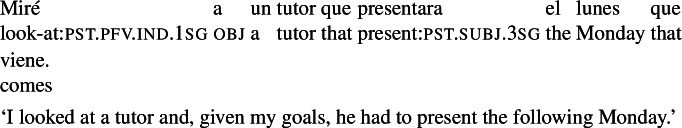

#### The circumstantial modal base: the role of diversity

As a starting point, we assume that a relevant fact that the circumstantial modal base yields when applied to a volitional event *e* is that *e* took place. We adopt David Lewis’ ontology (Lewis 1983, 1986) in which individuals are world-bound and cross-world identification amounts to a similarity relation between counterparts. Counterparts resemble each other closely, but they can do so in different ways; the similarity relation is vague. In the limit case, similarity can correspond to duplication. We assume that preserving the fact that the actual event took place amounts to saying that all worlds determined by the modal base contain a duplicate (an identical counterpart) of the actual event (including its actual participants). For instance, the modal base for subjunctive in our book example (67) will determine a set of worlds where an identical counterpart of the actual agent bought an identical counterpart of the actual book (at the time of the actual event).

(67)

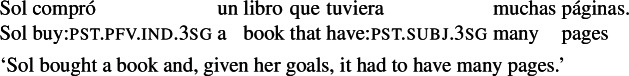

In (68) we show the truth conditions that our proposal currently delivers for (67) once we make explicit reference to counterparts (we assume that the function cp below maps an individual to the set of its counterparts and that an individual has only one counterpart in each world).

(68)

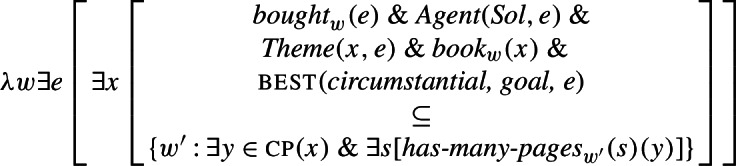

Note, first, that the assumption that the modal base contains duplicates of the actual event *e* predicts that when the property denoted by the RC temporally overlaps with *e*, this property will be true of the actual theme at the time of *e*. For instance, we predict (67) to entail that the book actually bought has many pages. The truth conditions in (68) state that the counterparts of the actual book *x* have many pages in the best worlds in the modal base. For this to be the case, the modal base has to contain *some* worlds where the counterparts of *x* have many pages. Since all counterparts of *x* throughout the modal base are identical, they *all* must have many pages. And since all of them are exact duplicates of *x*, *x* must also have many pages. This is in principle a welcome consequence: as noted in Sect. 2.3, the sentence in (67) does convey that the book bought has many pages.

But this way of determining the modal base also makes an incorrect prediction. In Sect. 2.3 we noted that (67) is false in the scenario in (69), where the agent bought a book with many pages, but not for that reason.

(69) ***Millenium*** **II.** Sol decided to buy her friend Marta a Swedish thriller, since she loves the genre. She found on the shelf the first book in the Millenium trilogy, so she bought that one. (But she would have preferred to buy a shorter book, as Marta easily gets tired of reading.)

Under our current assumptions, the truth conditions in (68) are incorrectly satisfied in the Millenium II scenario. We are assuming that the modal base worlds contain exact copies of the actual event, including its actual participants. In particular, the properties of the theme at the time of the event will be preserved. As noted above, given (69), that means that the agent bought a book with many pages across the set of worlds determined by the modal base (and therefore also in the best worlds in that set). More generally, as long as the book bought has many pages, (67) will come out true *regardless of what the agent’s goals were*.

We contend that the solution to this problem lies in an independently motivated requirement on modal bases: that they be *diverse* with respect to the prejacent (see Condoravdi 2002 and much following work). A modal base is said to be diverse with respect to a prejacent *p* iff the set of worlds determined by the modal base includes both worlds where *p* is true and worlds where *p* is false. We adopt the following formulation of the diversity condition, as an additional presupposition that is incorporated into the meaning of subjunctive mood.

(70)
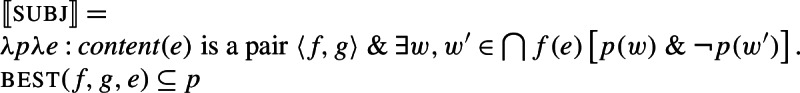

We propose that in cases like (69), the modal base is *expanded* to achieve diversity (i.e., to accommodate the second conjunct in the presupposition in (70)). This amounts to ignoring some propositions yielded by the original modal base *f* and using a modified $f'$.^33^ In our example (67), we need to weaken the similarity relation between counterparts so that the modal base includes both worlds where the counterpart of the actual book has many pages and worlds where it does not. If the agent’s goal was to buy a short Swedish thriller, the best among the expanded set of accessible worlds are ones in which the book she bought is not long. Once we have accommodated a diverse modal base, we no longer predict (67) to be true in the Millenium II scenario. More generally, we regain the intuition that agent-oriented RCs reflect the agent’s goals.

This move, though, raises another question, namely how to derive the intuition that the book bought had many pages. Once we allow expansion to achieve diversity, assuming a circumstantial modal base no longer automatically predicts actualization of the RC property. Suppose that the book bought, *x*, had few pages. The circumstantial modal base would first select a set of worlds where the counterparts of *x* also had few pages. This modal base would then be expanded to include worlds where the counterparts of *x* had many pages, thereby achieving diversity. The sentence in (67) could then come out true if the ordering source ranked as the best worlds in the expanded modal base those where the counterparts of *x* had many pages.

However, we argue that this situation is incompatible with the type of goals at play in agent-oriented RCs (Sect. 5.3.1). To see this, let us think about the kinds of situations where an agent *a* that wanted to buy a long book ends up buying a short one. One possibility is that buying a long book was not within *a*’s reach (suppose, e.g., that the only open store offered just short books, so *a* settled for a short book). In this case, buying a long book was not an action goal for *a*. Another possibility is that buying a long book was available to *a*, but conflicted with another goal of hers (for instance, imagine that *a* was getting ready for a long flight, and she wanted to buy a long book that would keep her entertained during the flight, but at the same time wanted to keep the weight of her carry-on light. She ended up deciding to buy a short book to satisfy the second goal.) In these cases, we would argue, *a*’s settling for a short book means that the action goal associated with the actual event is *not* that she buy a long book, so a sentence like (67) would come out false.^34^

Before closing this section, let us note that the modal bases associated with agent-oriented RCs with prospective orientation (at least those that describe non-scheduled events) satisfy the diversity condition to begin with, so there is no need for expansion. In that case, the worlds selected by the modal base still preserve the actual event, including the properties of the actual participants at the time of the event. But these worlds can be expected to differ with respect to how the participants evolve *after* the event time. (In line with much research on temporal-modal interactions, we are assuming a domain of possibilities where the past is fixed but the future is not deterministic, see discussion in Condoravdi 2002). Thus, the modal domain for, e.g., our radio example, will include both worlds where the counterpart of the radio (the theme of the buying event) entertains Pedro after the buying and worlds where it doesn’t (of those, the best worlds will be those where it does).^35^

### Subjunctive under subjunctive

Portner and Rubinstein’s (2020) analysis of subjunctive mood was developed to account for examples involving only one instance of subjunctive morphology, borne by a complement clause verb. Similarly, in the examples that we have focused on so far, there is only one subjunctive-marked verb, the one in the RC. As noted in Sect. 4, this raises the question of how multiple occurrences of subjunctive mood are interpreted. To address this question, we turn now to examples like (71), where a subjunctive RC (‘who lives in Northampton’) is embedded within the complement of a subjunctive-selecting verb (*querer*, ‘want’), so the complement clause verb (‘fall in love’) also bears subjunctive morphology.^36^

(71)

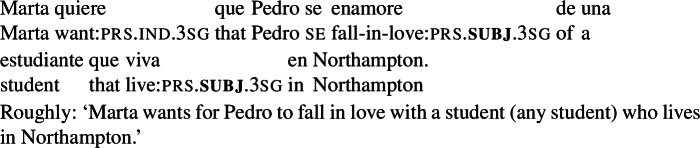

#### The data

If subjunctive morphology denotes a quantifier that is in the left periphery of the clause containing the subjunctive-marked verb, we would expect examples like (71) to involve two modals (as in (72)), and thus lead to the truth conditions in (73a). As proposed by Portner and Rubinstein (2020), the higher subj quantifier is anchored to the wanting event. And since the complement clause verb, *enamorarse* (‘fall in love’), is non-volitional, the only possible anchor for the lower subj would also be the wanting event.^37^^,^^38^ However, the interpretation in (73b), involving just one layer of modality (corresponding to the higher subjunctive), is available. To see that, consider the scenario in (74).

(72) [Marta wants_*e*_ [that subj_*e*_ Pedro falls in love with a student [that subj_*e*_ lives in Northampton]]]

(73)

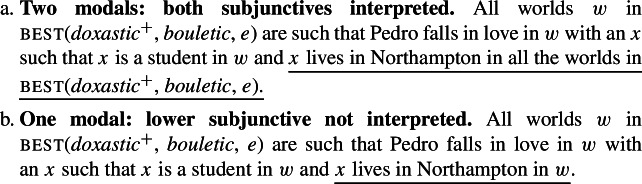

(74) The current students are *a*, *b* and *c*, and Marta is aware of that. Marta wants Pedro to fall in love with one of them—as far as she is concerned, any of the three would do. Marta wants whoever Pedro falls in love with to live in Northampton (so that they can all hang out together), but she wants all the other students to live in Amherst (otherwise there might be too many student parties in town).

Given (74), there are three types of worlds in best(*doxastic*^+^, *bouletic*, *e*), where *e* is the wanting event:^39^

(75)
  - worlds where Pedro falls in love with *a* and *a* lives in Northampton. *b* and *c* live in Amherst.
  - worlds where Pedro falls in love with *b* and *b* lives in Northampton. *a* and *c* live in Amherst.
  - worlds where Pedro falls in love with *c* and *c* lives in Northampton. *a* and *b* live in Amherst.

While the truth-conditions in (73b) (‘one modal’) are satisfied in this situation, the ones in (73a) (‘two modals’) are not—for the conditions in (73a) to be met, each of *a*, *b* and *c* should live in Northampton in *all* the worlds in best(*doxastic*^+^, *bouletic*, *e*). Thus, if (71) is judged as true in the scenario in (74), we will have evidence that the one modal interpretation is possible. This is indeed the case—speakers accept (71) in this context.

We have thus established that the lower subjunctive in examples with two instances of subjunctive marking does not have to be interpreted. But can it be? The example in (71) does not allow us to determine this: since (73a) (two modals) is logically stronger than (73b) (one modal), we cannot construct scenarios where (73a) is true and (73b) is false.^40^ To determine whether an interpretation with two modals is attested, we turn to examples like (76):

(76)

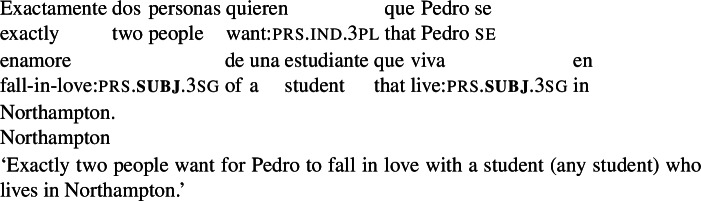

The interpretation of this example with two modals, (77a), does not entail the interpretation with one modal (77b). To see this, consider the scenario in (78).

(77)

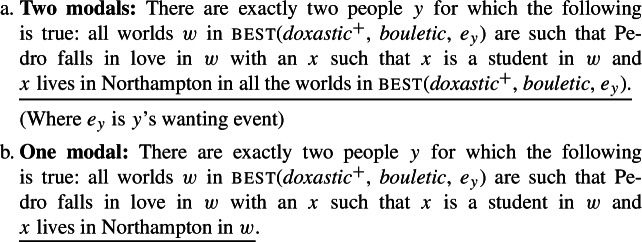

(78) The current students are *a*, *b*, and *c*, and Sara, Carla and Jonas are aware of that. Sara and Carla want all three students to live in Northampton. They furthermore want Pedro to fall in love with a student, any student of the three would do. Jonas, however, wants Pedro to fall in love with a student, any student, and he wants whoever Pedro falls in love with to live in Northampton, but any other students to live in Amherst.

In the scenario in (78) the reading with two modals is true, but the one modal reading is false, as there are *three* people whose desire worlds are such that in all of them Pedro falls in love with a student and that student lives in Noho. To our ear, the sentence is (76) is *false* in this scenario. According to our intuitions, then, the reading with two modals is not attested.

#### Double subjunctive as modal concord

The data in Sect. 5.4.1 raise two questions: How can we account for the one modal interpretation of our double subjunctive examples? Why is an interpretation with two modals not attested?

The one modal interpretation finds a parallel in modal concord, a phenomenon by which a sentence containing two modal elements is interpreted as involving only one layer of modality (see van Wijnbergen-Huitink 2021 for an overview). The example in (79), with the modal auxiliary *must* and the modal adverb *obligatorily*, provides an illustration. On its most salient reading, (79) conveys that students are under the obligation to register, not that it is necessary that they are under this obligation.

(79)

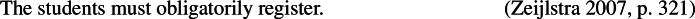

Zeijlstra (2007) puts forward an analysis of modal concord that assimilates it to negative concord, analysed as agreement in Zeijlstra 2004. While we do not commit to Zeijlstra’s analysis of examples like (79), we contend that his proposal can be extended to our double subjunctive examples. On this view, subjunctive morphology on a verb would signal agreement with a c-commanding quantifier subj (and, just as in other instances of concord, a single quantifier could license several instances of subjunctive morphology).

This amounts to adopting the second analytical option sketched in Sect. 5.1, where subjunctive *reflects* the presence of (rather than denotes) a quantifier in the left periphery of the clause. For cases like the ones discussed in Sects. 5.1 to 5.3, which involve just one instance of subjunctive morphology, nothing will change. For examples like (71), involving multiple instances of subjunctive morphology, we predict (80) to be a possible (simplified) structure. In this structure, agreement with a single subjunctive quantifier licenses subjunctive morphology both on the complement clause verb and on the RC verb.

(80) [Maria wants [that subj Pedro falls in love with [a student that lives in Northampton]]]

Why can’t we detect two modals? We are not sure. One possibility is that the absence of interpretations with two modals follows from economy considerations: perhaps subj is inserted only when required to license subjunctive morphology (one instance of subj is enough to license more than one instance of subjunctive morphology). Another possibility is that interpretations with two modals are in principle possible, but harder, in parallel to what happens in cases of negative concord, where double negation interpretations have been claimed to be restricted.^41^ Further empirical research is needed to determine whether interpretations with two modals are at all available, and under what conditions if so.^42^

### On the choice of determiner

The reader will have noticed that we have so far used indefinite DPs to illustrate our construction. The examples below show that other weak determiners (numerals, ‘many’, ‘several’) are possible, too.^43^

(81)

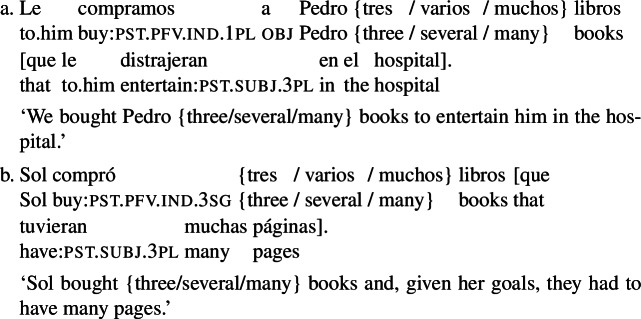

Strong, presuppositional, determiners, are also systematically possible with agent-oriented RCs that denote a property taken to be independently settled. For instance, the example in (82a), with the determiner *todos*, is fully acceptable and conveys, as before, that having many pages is the criterion that guided the agent when picking the books. In contrast, an example like (82b), with *todos* and an RC with prospective orientation is degraded, at least out of the blue.

(82)

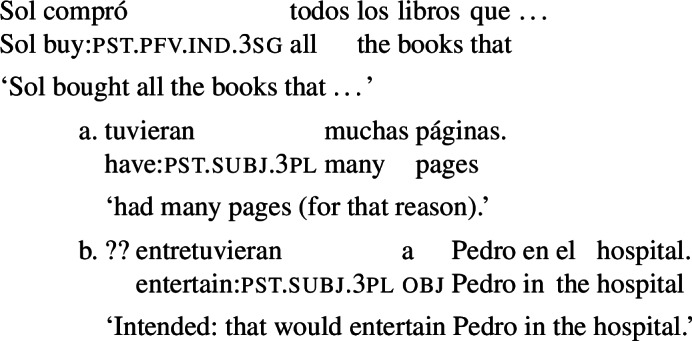

We believe that the oddity of examples like (82b) can be traced back to the way our analysis interacts with the pragmatics of presuppositional determiners. *Todos* presupposes that its domain is not empty. Given our account of agent-oriented RCs, the domain of *todos* in (82b) is the set of books that entertain Pedro in the hospital after the buying event, in all worlds satisfying the goals associated with the agent’s decision of buying. Furthermore, the RC would be redundant if all the books had the property that it denotes. This means that we would need to assume that, at the time of the decision, the books are of two kinds: ones that will entertain Pedro in the hospital in the worlds that fulfill the agent’s goals, and ones that will not. We contend that this condition is simply hard to accommodate, without further contextual clues.^44^

One way of rescuing examples like (82b) is to give the RC a dispositional or generic interpretation. The locative modifier ‘in the hospital’ in (82b) makes this difficult, but this possibility becomes available for the variation in (83), without the modifier. As the translation below indicates, (83) can be read as ‘Sol bought any books that normally entertain/could entertain Pedro.’ This interpretation is unproblematic as it is easy to imagine a situation where some books have the potential to entertain Pedro and some don’t (depending on what Pedro normally likes).

(83)

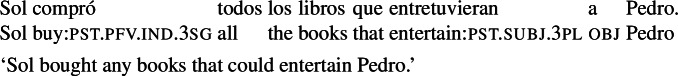

Future-oriented RCs that introduce a scheduled event are also good with *todos*, as illustrated by (84). If it is already decided who will perform in tomorrow’s show, the sentence is interpreted as saying that the agent interviewed all the actors that, in the worlds where her goals are satisfied, were (independently) scheduled to perform tomorrow. As the agent’s goals needs to be actionable, her goal worlds will not vary with respect to who performs. We will then understand that the agent interviewed all the actors that were actually scheduled to perform (as opposed to the ones that were not). As it is trivial to assume that not every actor will perform in the show, the split in the domain induced by the modifier will be easy to accommodate.

(84)

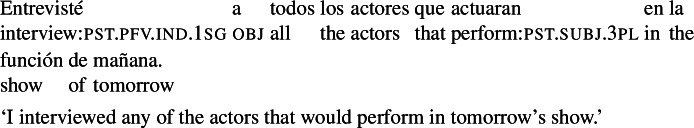

## Concluding remarks

Assuming that subjunctive mood is associated with a modal that can employ a projection mode available to modal indefinites explains the otherwise puzzling properties of agent-oriented subjunctive RCs. If our analysis is on the right track, agent-oriented RCs provide additional support for the Modal Anchor Hypothesis (Kratzer 2013) and for a decompositional approach to attitudes.

It is interesting to consider the difference between our account, on which modal anchors are events, with an alternative proposal where subj can be anchored to an individual. On this alternative view, the modality in agent-oriented RCs would be derived by accessing the intentions of the agent of the main event rather than this event itself. As noted by an anonymous reviewer, however, this would fail to predict the systematic ungrammaticality of examples like (24), as the external argument of a non-volitional verb like ‘discover’ can have action goals independently of their involvement in the event.

The next steps in this research program are to investigate whether our proposal can be successfully extended to other extensional environments where subjunctive RCs can occur, and whether it can derive the attested cross-linguistic variation in this domain. For instance, in Spanish (Rivero 1975) and Catalan (Quer 1998), subjunctive RCs are possible in subject position in examples like (85). In (85), the modality that subjunctive introduces seems to be epistemic (rather than teleological) and speaker- (rather than agent-) oriented: (85) conveys that the identity of the individual who did this varies across the speaker’s epistemic alternatives.

(85)

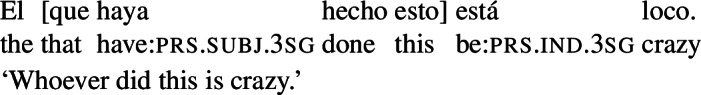

Turning to cross-linguistic variation, Farkas (1985, p. 156) reports that the configuration in (85) is not possible in Italian, Romanian, and French (which allow agent-oriented RCs, as noted in Sect. 2). At the same time, subjunctive RCs in extensional contexts in Romanian allow for modal flavours not attested in Spanish. For example, the Romanian construction in (86), where the RC has a deontic interpretation, is ruled out in Spanish.^45^

(86)

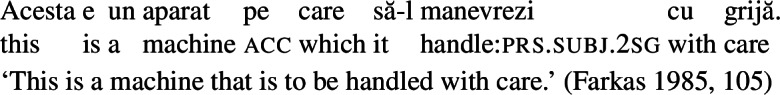

We hope to investigate the full empirical picture within Spanish and across Romance in future research.

## Footnotes

1 Some notable exceptions are Farkas 1985, Quer 1998, and Truckenbrodt 2019.

2 Italian *credere* (‘believe’) is a well-known exception, but see Mari and Portner (2021).

3 We use the following abbreviations in our glosses: ‘cond’: conditional, ‘impfv’: imperfective, ‘ind’: indicative, ‘obj’: differential object marker, ‘pfv’: perfective, ‘prs’: present, ‘pst’: past, ‘subj’: subjunctive, ‘sg’: singular, ‘pl’: plural, ‘se’: third person reflexive clitic.

4 To avoid clearly intensional environments, we set aside examples with verbs of creation (see Laca 2010). The assumption that transfer of possession verbs like *comprar* (‘buy’) and *enviar* (‘send’) are extensional is not universally shared (see Kratzer 2015 and Martin and Schäfer 2017 for an intensional analysis). However, the construction is not limited to transfer of possession verbs, see (3d).

5 Farkas (1985, Ch. 4) argues that, in the Romanian counterparts of the examples above, the RC is in an intensional context. While discussing cases where a free choice item is modified by a subjunctive RC, Chierchia (2013, Chap. 6) entertains the possibility that subjunctive may signal the presence of a modal operator taking scope over the RC.

6 It should be noted that this label is often applied rather broadly, and is intended to cover some cases where the RC is not in an extensional context. Quer (1998) remarks that purpose relatives in this broader sense occur under a subset of intensional predicates, future tense, and imperatives.

7 On subjunctive marking in purpose clauses, see Quer 2001.

8 This contrasts with what Farkas (1985) reports for Romanian. Farkas notes that (i) is ungrammatical in Romanian, as we would expect if agent-oriented RCs were necessarily linked to a purpose interpretation (Farkas 1985, p. 103). However, initial informal consultations reveal that at least some speakers accept (i) if the woman’s having blue eyes was the factor that led to the hiring. Further research is needed to determine the extent to which Romanian and Spanish pattern alike in this respect.

9 Past subjunctive morphology in Spanish has two allomorphs: *-ara* vs. *-ase* (for verbs whose infinitive ends in *-ar*) or *-iera* vs. *-iese* (for verbs whose infinitives ends in *-er* or *-ir*). In Peninsular Spanish, the *-ra* version is more common in spoken language (Alarcos Llorach 1994). Throughout the paper, we use this allomorph, but nothing hinges on this decision.

10 While the past subjunctive form that we are using here is labelled ‘imperfective subjunctive’ in descriptive grammars, aspect seems to be ‘fake’ as well (again, like in subjunctive conditionals, see Iatridou 2000). As shown by (i) below, this form is compatible with a perfective interpretation: the delivering event can be temporally included in the interval from three to five.

11 For subjunctive conditionals, see Iatridou 2000 and follow-up work. For the complement of modals, see Laca and Fălăuş 2020 and references therein.

12 Subjunctive RCs are possible in an external argument when in the scope of a subjunctive-selecting predicate, as in (i) below. We come back to this configuration in Sect. 5.4.

13 The construction is also possible when the main verb is in the present perfect form, witness (i) below.

We have opted for not using this form in our examples to avoid the well-known complications associated with the analysis of the present perfect.

14 For a general overview of modal indefinites, see Alonso-Ovalle and Menéndez-Benito 2020.

15 We come back to this example in Sect. 5.3.1. To make the right prediction, we need to assume that the modal domain is determined with respect to a *diverse* modal base (see Sect. 5.3.2).

16 For now, we gloss over how to represent cross-world identity. We come back to this in Sect. 5.3.

17 The behaviour of negation provides another parallelism with UC. To our ear, sentences like (i) are felicitous only when negation targets the RC (Juan *did* buy a book but not because it had many pages). While we cannot provide an explanation of this fact, we note that, as discussed by Alonso-Ovalle and Menéndez-Benito (2017) (see their fn. 26), negation also targets the agent-oriented component of UC.

18 Or events and their theme arguments (Kratzer 2006).

19 Note that modal backgrounds in what follows are functions from events to sets of propositions, rather than functions from worlds to sets of propositions as in Kratzer 1991.

20 Portner and Rubinstein (2020) assume that wanting relates to what they call a *doxastic*+ modal base, which provides an expansion of the belief-set of the attitude holder needed to account for well-known puzzles in the domain of belief and desire (see Heim 1992, von Fintel 1999, Rubinstein 2017).

21 This definition of the content of an attitude event is related to, but also differs from, the “content” that is familiar from Hacquard’s (2006, 2010) work. Hacquard (2010) associates content with both attitude and speech events, as do Portner and Rubinstein (2020), but she focuses on events that make accessible an (epistemic) information state and defines ⋂content(*e*) to be the doxastic alternatives of *e*’s attitude holder. The present definition applies to all kinds of attitudes, including ones that are not doxastic, and is sensitive, crucially, also to ordering sources.

22 Their account thus belongs to theories that relate the subjunctive to modal comparison (Giorgi and Pianesi 1997, Villalta 2006, 2008, Giannakidou and Mari 2021, among others). See Kratzer 1981 for the definition of the world ordering relation.

23 This is a simplified analysis, which glosses over the question of how to model the weaker necessity of the subjunctive. We refer the reader to Portner and Rubinstein 2020 for a proposal based on Kratzer’s (1991) Good Possibility relation (see Portner and Rubinstein 2016, 2020, Rubinstein 2020).

24 Portner and Rubinstein’s analysis raises the question of how to best analyze mood in root clauses. As is well known, the canonical mood of root declaratives and interrogatives in Romance is the indicative. Portner and Rubinstein (2020) suggest in passing (see their fn. 53) that root indicative is anchored to the utterance situation, whose content would give the common ground.

25 Portner and Rubinstein (2020) do not assume that event arguments are syntactically represented. As shown in Sect. 4, in their system attitude verbs and their complements combine by Predicate Modification. This is crucial, as the argument of mood in complement clauses must be identified with the attitude event. To derive the same result in a system where event arguments are represented in the syntax, we need to make a further assumption. The LF in (47a), repeated below in (i), should be updated as in (ii), where event arguments are represented as subscripts (world arguments are omitted). That way, nodes  and  will still combine by Predicate Modification, yielding the desired result.

26 However, *promise* does not license subjunctive in the complement clause, a well-known puzzle for theories of mood selection (but see Portner and Rubinstein 2012).

27 We remain agnostic with respect to how to derive the correlation between different aspectual classes and temporal orientation (for a recent overview of the topic, see Laca and Fălăuş 2020).

28 As an anonymous reviewer notes, this could be derived by assuming that the Voice head introduces existential closure.

29 It is worth asking *why* the modal anchor of the subjunctive has to remain under the scope of the existential closure operator. We only have a speculation to offer. Percus (2000) observes that some world variables need to be locally bound. von Fintel and Heim (1997–2021, p. 94) explore the possibility that world variables slots are filled by a covert operator (OP). OP moves, leaves behind a world variable and derives, as a result of movement, a property of worlds. The operator is otherwise semantically vacuous. We could treat the modal anchor of subjunctive RCs in a similar way. The modal anchor slot could be filled by a covert operator (OP_ev_) whose movement would derive a property of events. That property would combine with Agent in (51) and feed the existential closure operator. Under this view, there would be no need to stipulate independently the insertion of a covert lambda abstracting over the modal anchor. We could hypothesize, like von Fintel and Heim do for OP, that the movement of OP_ev_ is local. In any case, moving OP_ev_ over the existential closure operator would derive a type different than that of a proposition, resulting in a non-assertable semantic object.

30 We are abstracting away from the details of Alonso-Ovalle and Menéndez-Benito’s implementation, where ‘action goals’ are characterized as sets of possible events rather than propositions.

31 The relation between the matrix agent and the RC is reminiscent of Farkas’s resp relation (Farkas 1998, 1992a), relevant for canonical control constructions and obviation, as characterized by Szabolcsi (2021). According to Szabolcsi (2021), who focuses on obviation in Hungarian under desiderative verbs, the subject does not stand in a resp relation to the complement situation when “this individual either does not intentionally bring about that situation at all, or he/she needs the authority or cooperation of others to bring it about” (p. 7).

32 This raises the question of why our radio example is fine. Somehow here the fact that the radio is designed (among other things) to entertain people seems to be enough. Further research is needed to establish what types of situations count as being within the control of the agent for the purposes of our construction.

33 A similar operation has been motivated for the doxastic-oriented modal base of desire predicates (see Villalta 2008, Rubinstein 2017, Grano and Phillips-Brown 2022 on *want*).

34 Situations where the agent was misinformed provide an interesting borderline case. Suppose that Sol wanted to buy a long book, and selected one at Amazon. However, the description of the book at Amazon was wrong, and the book bought turned out to actually be short. Judging examples like (67) in this kind of situation is not easy. We believe that this difficulty has to do with the fact that the agent thought her goal of buying a long book was an action goal, but it actually was not (as the agent lacked crucial information). Given the actual circumstances, the sentence is false, but switching to the perspective of the agent might allow for a true judgment.

35 On the connection between future-orientation and diversity, see Condoravdi 2002.

36 Unlike in the rest of the paper (see Sect. 2.5), our examples in this section have present-marked main verbs. This is to avoid additional complications that might arise when interpreting perfective morphology on the stative verb ‘want’.

37 As noted in Sect. 4, in this case the modal domain will consist of the worlds selected by a (subtype) of doxastic modal base (*doxastic*+) that are best with respect to a bouletic ordering source, corresponding to the agent’s desires.

38 As discussed in Sect. 5.1, mood can be anchored non-locally, i.e., to an event in a higher clause.

39 For simplicity, we are again glossing over the issue of cross-world identity, and tacitly assuming a counterpart relation of total similarity.

40 Given (73a), all worlds in the domain will have a student who Pedro falls in love with and who lives in Northampton. For (73b) to be *false*, there must be at least one world that doesn’t have any such student. Thus, if (73a) is true, (73b) must be true.

41 Zeijlstra (2008, fn. 1) and Chierchia (2013, 229) report that double negation readings are generally not possible in Italian. The former notes that they depend on marked prosody. Espinal et al. (2016) show experimentally that double negation readings in Catalan are rejected with a default prosody, and only accepted when the negative word is realized with a marked contour.

42 An additional note is in order: both the complement clause and the RC verbs have to be interpreted in the desire worlds (not $w_{0}$). We take this to follow from Percus’s (2000) Generalization X (world pronouns must be bound by the closest *λ* binder).

43 Pérez Saldanya (1999) also provides an example with *unos cuantos* (‘a few’), reproduced above in fn. 13.

44 Agent-oriented RCs seem degraded with definite DPs across the board, as illustrated by (i) below.

This seems to be an instance of a more general restriction: Pérez Saldanya (1999) notes that, under intensional verbs like *search*, definite DPs containing a subjunctive RC (as in (ii) below) are unacceptable. We have to leave this as an issue for further research.

45 We are grateful to Donka Farkas (p.c.) for directing us to this example. See Farkas (1985) for contrasts between French/Italian and Romanian subjunctive RCs.
